# Single‐cell protein profiling defines cell populations associated with triple‐negative breast cancer aggressiveness

**DOI:** 10.1002/1878-0261.13365

**Published:** 2023-01-25

**Authors:** Barbora Kvokačková, Radek Fedr, Daniela Kužílková, Jan Stuchlý, Adéla Vávrová, Jiří Navrátil, Pavel Fabian, Róbert Ondruššek, Petra Ovesná, Ján Remšík, Jan Bouchal, Tomáš Kalina, Karel Souček

**Affiliations:** ^1^ Department of Cytokinetics Institute of Biophysics of the Czech Academy of Sciences Brno Czech Republic; ^2^ International Clinical Research Center St. Anne's University Hospital Brno Czech Republic; ^3^ Department of Experimental Biology, Faculty of Science Masaryk University Brno Czech Republic; ^4^ Childhood Leukaemia Investigation Prague Czech Republic; ^5^ Department of Pediatric Haematology and Oncology, 2nd Faculty of Medicine Charles University Prague and University Hospital Motol Czech Republic; ^6^ Faculty of Science Charles University Prague Czech Republic; ^7^ Department of Comprehensive Cancer Care Masaryk Memorial Cancer Institute Brno Czech Republic; ^8^ Department of Oncological Pathology Masaryk Memorial Cancer Institute Brno Czech Republic; ^9^ Department of Clinical and Molecular Pathology, Institute of Molecular and Translational Medicine, Faculty of Medicine and Dentistry Palacký University and University Hospital Olomouc Czech Republic; ^10^ Department of Pathology EUC Laboratoře CGB a.s. Ostrava Czech Republic; ^11^ Institute of Biostatistics and Analyses, Faculty of Medicine Masaryk University Brno Czech Republic; ^12^ Human Oncology and Pathogenesis Program Memorial Sloan Kettering Cancer Center New York City NY USA

**Keywords:** mass cytometry, phenotypic plasticity, single‐cell profiles, triple‐negative breast cancer, tumor heterogeneity, unsupervised machine learning algorithm

## Abstract

Triple‐negative breast cancer (TNBC) is an aggressive and complex subtype of breast cancer that lacks targeted therapy. TNBC manifests characteristic, extensive intratumoral heterogeneity that promotes disease progression and influences drug response. Single‐cell techniques in combination with next‐generation computation provide an unprecedented opportunity to identify molecular events with therapeutic potential. Here, we describe the generation of a comprehensive mass cytometry panel for multiparametric detection of 23 phenotypic markers and 13 signaling molecules. This single‐cell proteomic approach allowed us to explore the landscape of TNBC heterogeneity, with particular emphasis on the tumor microenvironment. We prospectively profiled freshly resected tumors from 26 TNBC patients. These tumors contained phenotypically distinct subpopulations of cancer and stromal cells that were associated with the patient's clinical status at the time of surgery. We further classified the epithelial‐mesenchymal plasticity of tumor cells, and molecularly defined phenotypically diverse populations of tumor‐associated stroma. Furthermore, in a retrospective tissue‐microarray TNBC cohort, we showed that the level of CD97 at the time of surgery has prognostic potential.

## Graphical abstract

The extensive heterogeneity of triple‐negative breast cancer (TNBC) affects disease progression and therapeutic response. To investigate TNBC heterogeneity in more detail, we profiled 26 treatment‐naïve primary TNBC at the single‐cell level using mass cytometry. We introduced a new clinically relevant Ki‐67+LNR index and identified distinct tumor and stromal populations associated with clinical observations.

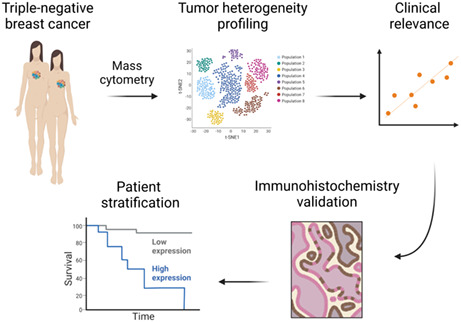

**Abbreviations**
CAF: cancer‐associated fibroblasts
CSC: cancer‐stem cells
E/M: epithelial‐mesenchymal
EMT: epithelial‐mesenchymal transition
IHC: immunohistochemistry
LNR: lymph node ratio
MET: mesenchymal‐epithelial transition
PBMCs: peripheral blood mononuclear cells
TMA: tissue microarray
TME: tumor microenvironment
TNBC: triple‐negative breast cancer
t‐SNE: t‐distributed stochastic neighbor embedding

## 1 Introduction

Triple‐negative breast cancer (TNBC) is a profoundly heterogeneous subtype of breast cancer with characteristic aggressive behavior and poor outcome [1, 2]. Because TNBC lacks the expression of estrogen (ER), progesterone (PR), and human epidermal growth factor 2 (HER2) receptors, cytotoxic chemotherapy remains the treatment of choice for early‐stage and advanced TNBC. However, multiple clinical trials with targeted approaches and immunotherapies are ongoing [3].

Intra‐ and intertumoral heterogeneity are major obstacles in the effective clinical management of patients. Both were in part molecularly dissected by a number of studies that identified multiple TNBC subtypes, with each exhibiting unique biological features [4]. The intratumoral heterogeneity is driven by genetic, epigenetic, and phenotypic inputs within the cancer cells and extrinsic factors from the tumor microenvironment (TME). Such heterogeneity can then influence tumor progression and therapeutic response [5]. Phenotypic plasticity of TNBC is often attributed to epithelial‐mesenchymal transition (EMT), a mechanism that generates a subpopulation of highly motile cells, often with increased ability to seed new tumors and capacity to self‐renew and differentiate [6, 7]. EMT and its reverse program mesenchymal‐epithelial transition (MET) are key advantageous developmental programs hijacked by tumor cells to support their dissemination from the primary site. While EMT and acquisition of mesenchymal phenotype (M) is critical for cancer cell spread, subsequent reversion back to the epithelial state (E) is a prerequisite for successful metastatic outgrowth at distant sites [5, 8, 9].

Selected tumor cell populations can dynamically switch between EMT and MET and exist in a spectrum of hybrid E/M states, partially bearing features of both phenotypes. Cells in these hybrid states are presumed to have the highest tumorigenic and metastatic potential [10, 11]. Both EMT/MET programs are influenced by a number of factors and signaling molecules, including the stereotypical EMT inducer TGF‐β and SMAD signaling pathways, NF‐κB, JAK–STAT proteins, PI3K/AKT/mTOR or Wnt [5, 12]. These stimuli are usually governed by TME, comprised of different populations of infiltrating immune cells, stromal cells such as fibroblasts, pericytes, endothelium, and other cell types together with extracellular matrix [13]. Various elements of TME play both pro‐ and anti‐tumor roles, and TME represents a promising therapeutic target [13, 14]. An ample effort is currently being made toward the detailed characterization of tumor‐TME interactions at single‐cell and spatial levels. Several recent single‐cell transcriptomic reports further confirmed that TNBC tumors consist of distinct subpopulations of tumor cells [15, 16], cancer‐associated fibroblasts [17], and immune cells [18] with clinically relevant transcriptomic signatures. The observed gene expression changes are not always reflected at the level of protein expression. Mass cytometry overcomes this limitation by employing metal‐conjugated antibodies, enabling the quantification of dozens of proteins and phospho‐epitopes in individual cells simultaneously [19].

In this study, we applied such mass cytometric profiling to a cohort of prospective TNBC patients. We comprehensively mapped phenotypic TNBC diversity and signaling status at the protein level. Our data revealed the presence of distinct cancer and stromal phenotypes within TNBC tumors, and their association with patient clinical status. Validation of selected markers in expanded retrospective cohorts with routine histology techniques further corroborated their stratifying potential.

## 2 Materials and methods

### 2.1 Cell lines

Breast cancer cell line MDA‐MB‐231 (RRID:CVCL_0062) was obtained from the American Type Culture Collection (ATCC, Manassas, Virginia, USA) and used as an internal control. Cells were cultured in RPMI 1640 medium (Thermo Fisher Scientific, Waltham, Massachusetts, USA, TFS) supplemented with 10% fetal bovine serum (TFS) and 100 U·mL^−1^ penicillin/streptomycin (Sigma‐Aldrich, Merck‐Millipore, Darmstadt, Germany). Cells were maintained at 37 °C and 5% CO_2_, routinely tested for mycoplasma contamination with PCR and authenticated using AmpFLSTR Identifiler Plus PCR Amplification Kit (TFS) to verify their origin. Peripheral blood mononuclear cells (PBMCs) were obtained from a healthy donor with written informed consent in accordance with the Declaration of Helsinki. PBMCs were isolated as a buffy coat layer on Ficol‐Paque gradient. Isolated PBMCs and the MDA‐MB‐231 cell line were incubated with CD24 and ROR1 antibodies for 30 min at room temperature (Table 1). After washing once with Maxpar Cell Staining Buffer (CSB) (Fluidigm, South San Francisco, CA, USA), cells were stained with 1 μm cisplatin for subsequent dead cell exclusion (Fluidigm) and quenched with CSB buffer. Cells were then fixed with 1.6% paraformaldehyde (TFS) for 15 min at room temperature and stored at −80 °C in 10% glycerol in fetal bovine serum.

**Table 1:** Overview of antibodies and reagents used in study.

Mass cytometry				
Marker	Clone	Metal	Source	Cat. no; home‐made LOT identifier
CD45	HI30	Y89	Fluidigm	3089003B
CD28	CD28.2	142Nd	Bxcell	BE0291; 180116
CD49f	GoH3	143Nd	Biolegend	313602; 180906
CD69	FN50	144Nd	Fluidigm	3144018B
CD4	RPA‐T4	145Nd	Fluidigm	3145001B
CD8a	RPA‐T8	146Nd	Fluidigm	3146001B
ITGB5	AST‐3 T	147Sm	Biolegend	345202; 180919
CD111	R1.302	148Nd	Biolegend	340402;180906
CD38	HIT2	149Sm	Exbio	11‐366‐C100, 190425
CD112	TX31	150Nd	Biolegend	337402; 180919
EpCAM	9C4	152Sm	Biolegend	324229; 180919
CD29	TS2/16	156Gd	Fluidigm	3156007B
ROR‐1	2A2	159Tb	Miltenyi Biotec	130‐98‐243; 191209
CD14	M5E2	160Gd	Fluidigm	3160001B
CD49c	ASC‐1	162Dy	Biolegend	343802; 180906
CD24	ML5	165Ho	Biolegend	311127; 181129
CD44	BJ18	166Er	Fluidigm	3166001B
CD90	5E10	167Er	Biolegend	328129; 180919
CD19	HIB19	169Tm	Fluidigm	3169011B
CD3	UCHT1	170Er	Fluidigm	3170001B
CD97	VIM3b	171Yb	Fluidigm	3171015B
CD9	SN4_C3‐3A2	172Yb	Fluidigm	3172010B
HLA‐DR	L243	174Yb	Fluidigm	3174001B
CD31	MEM‐05	175Lu	Exbio	11‐273‐C100; 190516
CD56 (NCAM)	NCAM16.2	176Yb	Fluidigm	3176008B
αSMA	1A4	141Pr	Fluidigm	3141017D
pSmad1 (Ser463/465)/ Smad5 (Ser463/465)/ Smad9 (Ser465/467)	D5B10	151Eu	Cell Signaling	#12428; 191209
Stat1 (Y701)	58D6	153Eu	Fluidigm	3153003A
Vimentin	D21H3	154Sm	Fluidigm	3154014A
pSmad2 (Ser465/Ser467)	E8F3R	155Gd	Cell Signaling	#18338; 181129
pStat3 (Y70)	4/P‐STAT3	158Gd	Fluidigm	3158005A
pNF‐kBp65 (S536)	93H1	161Dy	Cell Signaling	#3033;170313
pAkt (S473)	D9E	163Dy	Cell Signaling	#4060; 170313
Active β‐catenin (Ser45)	D2U8Y	164Dy	Cell Signaling	#19807; 181129
Ki‐67	B56	168Er	Fluidigm	3168007B
PanCK	C‐11	173Yb	Exbio	11‐108‐C100; 180919
CD298	REA217	N/A	Miltenyi	130‐122‐331; N/A
HLA‐I	W6/32	N/A	Bxcell	BE0079; N/A
Chemicals				
Platinum (^195^Pt, ^196^Pt)			Fluidigm	201195, 201196
Cadmium (^106^Cd, ^110^Cd, ^111^Cd, ^112^Cd, ^113^Cd, ^114^Cd and ^116^Cd)			Fluidigm	N/A
CisPt		198Pt	Fluidigm	201198
Iridium (^191^Ir, ^193^Ir)			Fluidigm	201192A

Immunohistochemistry				
Marker	Clone	Host	Source	Cat. no
EpCAM	Ber‐Ep‐4	Mouse	DCS	EI751C002
CD49f	Polyclonal	Rabbit	Invitrogen	PA5‐82466
Vimentin	SP20	Rabbit	DCS	VI686C002
CD90	D3V8A	Rabbit	Cell Signaling	13801
αSMA	1A4	Mouse	Dako	M0851
HLA‐DR	TAL. 1B5	Mouse	Dako	M0746
NF‐kB p65	F‐6	Mouse	Santa Cruz Biotechnology	sc‐8008
CD49c (ITGA3)	polyclonal	Rabbit	Sigma‐Aldrich	HPA008572
CD97	EPR4427	Rabbit	Abcam	ab108368
AR	AR 441	Mouse	Dako	M3562
CK5/6	D5/16 B4	Mouse	Roche	790‐4554
E‐cadherin	NCH‐38	Mouse	Dako	M3612
CD8	SP16	Rabbit	Thermo Fisher Scientific	RM‐9116‐S1

### 2.2 Patient samples

Fresh breast cancer tissues from patients undergoing surgical breast cancer removal, in excess of that required for diagnostic and therapeutic procedures, were obtained from Masaryk Memorial Cancer Institute (MMCI) between 2018 and 2021 (Table S1). Tissues were evaluated by a certified breast cancer pathologist. The study was approved by the Ethical Committee of the MMCI (Ref. No. 2017/1894/MOU). The experiments were conducted with the understanding and written consent of each patient.

### 2.3 Breast cancer tissue processing

Tissue samples were minced to 1–2 mm pieces and enzymatically digested in DMEM/F12 (Gibco, TFS) containing 2% bovine serum albumin (BSA; Serva, Heidelberg, Germany), 5 mg·mL^−1^ recombinant human insulin (Sigma‐Aldrich), 0.5 mg·mL^−1^ hydrocortisone (Sigma‐Aldrich), 50 mg·mL^−1^ gentamicin (Serva), 2 mg·mL^−1^ collagenase type I (cat. no. LS 004194; Worthington, Lakewood, NJ, USA), 0.6 U·mL^−1^ dispase II (cat. no. 04942078001; Roche, Basel, Switzerland) and 10 mm Y‐27632 dihydrochloride (ROCK inhibitor, Santa Cruz Biotechnology, Dallas, TX, USA), for 14 h at 37 °C using 10 rpm agitation. Samples were then treated with 15 mg·mL^−1^ DNase I (cat. no. 04942078001; Roche) for 5 min at 37 °C, washed with PBS, and filtered through a 70 μm strainer. Red blood cells were lysed with ACK buffer (155 mm ammonium chloride, 10 mm potassium bicarbonate, and 100 μm EDTA solution in sterile MQ water) at 37 °C for 5 min, washed with PBS and incubated with CD24 and ROR1 antibodies 30 min at room temperature. After washing once with CSB buffer, cells were stained with 1 μm cisplatin for subsequent dead cell exclusion (Fluidigm) and quenched with CSB buffer. Cells were then fixed with 1.6% paraformaldehyde (TFS) for 15 min at room temperature and stored at −80 °C in 10% glycerol in fetal bovine serum.

### 2.4 Mass cytometry barcoding

To achieve parallel sample analysis and thus minimize the batch effect, we used metal tagged antibody‐based barcoding approach. 0.1–1 × 10^6^ cells from each dissociated tumor sample, as well as MDA‐MB‐231 cells and PBMCs (internal controls) were barcoded using a barcoding scheme consisting of combinations of HLA‐I and CD298 antibodies conjugated to platinum (^195^Pt and ^196^Pt) and cadmium (^106^Cd, ^110^Cd, ^111^Cd, ^112^Cd, ^113^Cd, ^114^Cd, and ^116^Cd) isotopes (see Table 1). Antibodies were purchased from Fluidigm or conjugated in‐house using Maxpar antibody conjugation kits (Fluidigm). Thawed individual tumor samples, cell line, and PBMCs were incubated with antibodies for 30 min at room temperature, washed with CSB buffer, and pooled together for subsequent antibody staining.

### 2.5 Antibodies and antibody labeling

All antibodies, clones, metal tags, and providers are listed in Table 1. Metal‐labeled antibodies were prepared using the Maxpar antibody conjugation kits (Fluidigm), according to the manufacturer's instructions or purchased pre‐conjugated. Each antibody was titrated and validated into the working panel to achieve an optimal signal‐to‐noise ratio.

### 2.6 Mass cytometry antibody staining and detection

After barcoding, the pooled samples were stained with a mastermix of surface antibodies for 30 min at room temperature; except for CD24 and ROR1 that were stained before fixation and freezing at −80 **°**C. Samples were then washed twice with CSB buffer. Next, the cells were permeabilized with 80% methanol for 30 min on ice and washed with CSB buffer. For intracellular staining, the cells were incubated with a mastermix of intracellular antibodies for 30 min at RT and then washed twice with CSB buffer. Lastly, samples were resuspended in 1 mL of Cell‐ID Intercalator‐Iridium in MaxPar Fix & Perm Buffer (Fluidigm). Cells were then washed twice with CSB buffer, once MiliQ H_2_O, resuspended in 20% EQ Four Element Calibration Beads (Fluidigm) in MiliQ H_2_O, and filtered through a 40 μm filter cap. Sample analysis was acquired on Helios™, a CyTOF® system (Fluidigm) with all opened channels and data collected as .fcs files.

### 2.7 Data preprocessing and analysis

Data were normalized in fluidigm software based on EQ beads and compensated for channel crosstalk, as previously described [20]. These compensated .fcs files were processed with flowjo software (v10.8.0, BD). Cells were gated for singlets, de‐barcoded, and cisplatin‐positive dead cells were excluded from the analysis. The following major cell types of interest were identified by manual gating using selected marker expression pattern: PanCK^+^/CD45^−^ cancer cells, CD45^+^PanCK^−^ immune cells, CD90^+^/PanCK^−^/CD45^−^ stromal cells. Representative gating strategy is shown in Fig. S1A. The total amount of collected cells from measured samples ranged from 850 to 174 000. The resulting, clean dataset contains in total 73 532 cancer cells, 560 218 immune cells, and 156 928 stromal cells.

For the purposes of unsupervised data analysis, we utilized recently published Risk Assessment Population IDentification (RAPID) algorithm [21], enabling identification of phenotypically distinct populations and determining whether they stratify patient survival. Twenty‐six manually pre‐gated .fcs files were exported, and loaded into rstudio (4.1.1 version) [22]. Here, each file was downsampled to the same cell number and combined into new data frame. Because some samples contained lower number of cells they were excluded for further unsupervised analysis of epithelial cells (21 samples analyzed in total) and stromal fraction (24 samples analyzed in total). This preprocessed mass cytometric data frame, together with .csv file containing annotated clinical parameters of tumor specimens, was subjected to a modified RAPID algorithm. Dimensionality reduction was calculated based on 36 markers using the r t‐sne package [23]. The following parameters were used: iteration = 10 000, perplexity 200, theta 0.9, and eta 200. The optimal number of clusters was computed based on the t‐SNE map using the modified script, followed by FlowSOM clustering [24].

Because our dataset lacked information about the patient outcome, on which the RAPID algorithm is based, we introduced a Ki‐67 + LNR index that consisted of clinical assessment of cancer spread to lymph nodes and Ki‐67 positivity, as determined by diagnostic histology analysis for each patient. This clinically relevant Ki‐67 + LNR index was then implemented for modified RAPID analysis instead of patient survival.

To calculate Ki‐67 + LNR index for each patient we used two variables, LNR (“lymph node ratio” = number of positive lymph nodes over number of excised lymph nodes) and Ki‐67 that was normalized to maximum value from all tumors. For each analyzed cell in the dataset, we stored also information from what tumor/patient the cell originated and the cluster number in which the cell was identified by FlowSOM. We took normalized Ki‐67 and LNR values of single tumors and assigned them to each analyzed cell in dataset as new variables. Then we selected cells of separate FlowSOM clusters and calculated the means of normalized Ki‐67 and LNR variables. Finally, a sum of these two means results in Ki‐67 + LNR index that acquires values from 0 to 2.

Mesenchymal‐epithelial (MET) score was calculated for each cancer cell. Mean intensity (MI) of selected proteins measured by mass cytometry was normalized to their corresponding maximal values obtained in the dataset. The normalized values of the final range from 0 to 1 were then used to calculate the MET score. The mass cytometry MET score was then calculated as a cumulative sum of normalized MIs from epithelial markers [EpCAM + CD49f + CD9] minus the cumulative sum of MIs from mesenchymal markers [Vimentin + αSMA + CD44] for each cancer cell in the sample. The final MET score for each patient is calculated as the mean MET score from all cancer cells in the sample and acquires values from −3 (fully mesenchymal‐like phenotype) to +3 (fully epithelial‐like phenotype). MET score calculation was done in r.

Correlation analysis was performed in graphpad prism (version 9.0.2 GraphPad Software, San Diego, CA, USA) using non‐parametric Spearman's correlation. Heatmaps were generated in the clustvis tool [25].

### 2.8 Tissue microarray (TMA) construction and immunohistochemistry

The first set of archival tissue samples from TNBC patients was constructed at the MMCI, and contained patient tissue collected between 2005 and 2009. The second set was also constructed at the MMCI, collected between 2012 and 2021. TNBC status was determined according to ASCO standards – threshold for the hormone receptor for IHC staining of tissue sample was 1% [26]. TMA construction and immunostaining of archival formalin‐fixed, paraffin‐embedded tumor samples with appropriate antibodies was done according to standard techniques established at MMCI (Table 1). Protein expression was assessed semi‐quantitatively by an expert breast cancer pathologist, using the histoscore (H‐score) method. In H‐score, the percentage of positive cells (0–100%) is multiplied by staining intensity (0–3), resulting in a final histoscore that ranges between 0 and 300. Immunohistochemistry MET score was calculated as a cumulative H‐score of [EpCAM + CD49f] proteins minus a cumulative H‐score of [Vimentin + αSMA] proteins. For Burstein classification of tumor tissues we used five surrogate markers – AR, CK5/6, CD8, E‐cadherin, Vimentin – in co‐junction with quantification of tumor‐infiltrating lymphocytes according to the relevant studies [27, 28, 29]. We then classified these samples according to Burstein's classification as luminal‐androgen receptor (LAR), mesenchymal (MES), basal‐like immunosuppressed (BLIS), and basal‐like immunoactivated (BLIA) subtypes. TNBC subtypes for this cohort were added to Table S1 and used for further analysis.

### 2.9 Statistical analysis

Statistical analyses and visualizations were performed in r (4.1.1 version, R Core Team, 2021) and graphpad prism (version 9.0.2, GraphPad Software). Kaplan‐Maier analysis was performed by the survminer r package. Illustrations were created with BioRender.

## 3 Results

### 3.1 Broad view of TNBC cellular landscape through large‐scale single‐cell proteomics

**Fig. 1:**
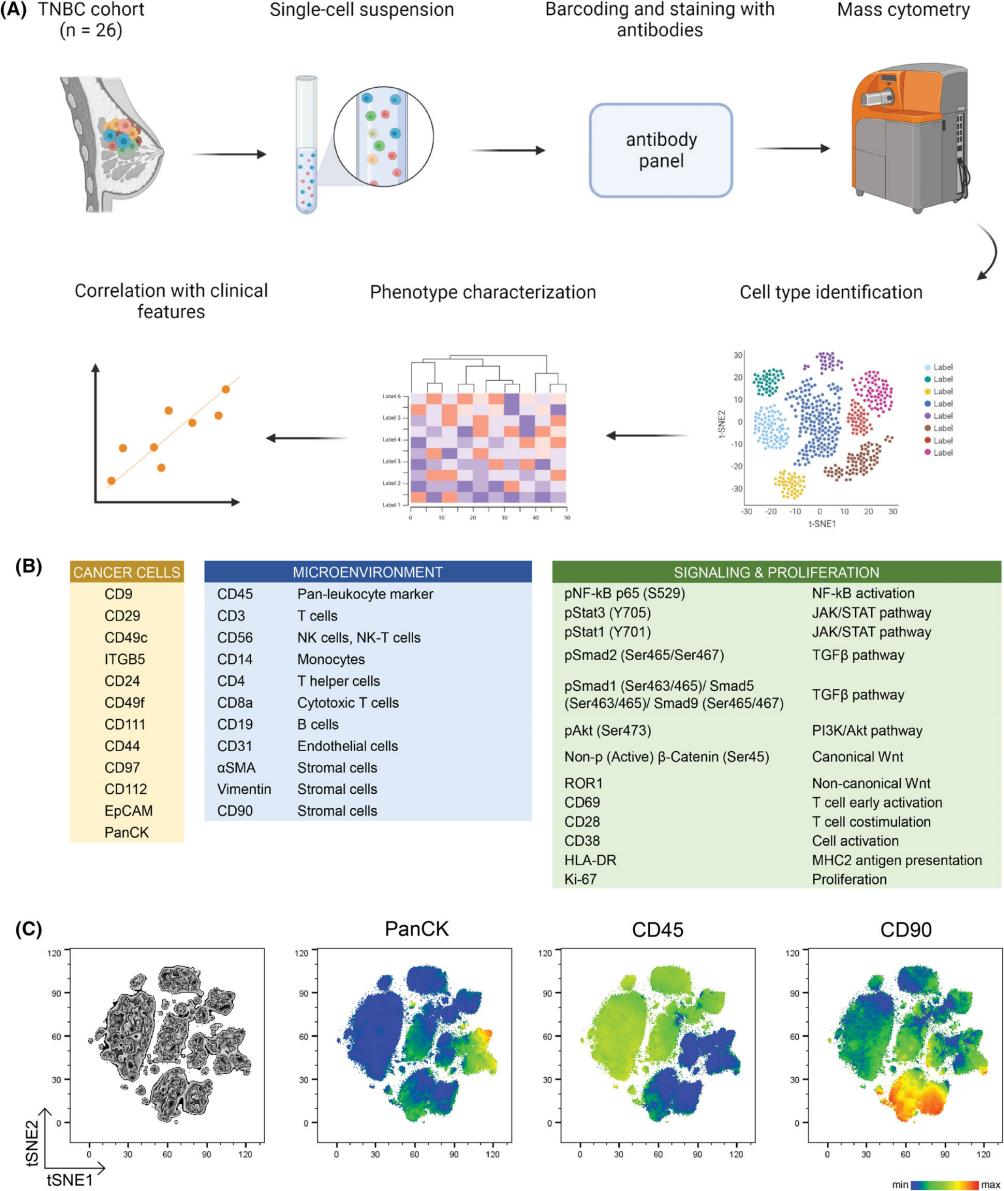
Characterization of tumor heterogeneity in TNBC samples by mass cytometry. (A) Scheme depicting experimental approach and analytical workflow for primary TNBC patient samples used in this study. (B) List of cell surface and signaling molecules selected to characterize tumor and microenvironmental compartments. (C) Two‐dimensional t‐SNE visualization of PanCK, CD45, and CD90 expression in all cells and all samples (*n* = 26). The combination of these three markers was used for the identification of cancer (PanCK+), immune (CD45+), and stromal (PanCK‐CD90+) cells, respectively.

While single‐cell transcriptomics vastly expanded our understanding of tumor ecosystems, this approach does not yet allow plausible profiling of cell landscapes in larger patient cohorts or greater cell numbers. To challenge this limit, we designed a prospective, single‐cell‐based, large‐scale mass cytometric analysis of 26 treatment‐naïve triple‐negative breast cancer cases. Twenty‐four tumors were classified as high grade 3, and two were intermediate grade 2 tumors. Six patients presented with involved lymph nodes that have been resected, and none of them had signs of metastatic disease at the time of surgery (Table S1). Freshly resected tumor samples were immediately dissociated into single cell suspensions and mass‐tag barcoded, along with a cancer cell line and PBMCs that served as internal controls. Samples were then sequentially stained with a panel of surface and intracellular antibodies (Fig. 1A).

The antibody panel was designed to identify tumor cell subpopulations that are known to contribute to breast cancer progression (e.g. CD24, CD44 as markers of CSCs), surface molecules that associate with EMT/MET plasticity (e.g. EpCAM and Vimentin) and a set of novel surface antigens reflecting breast cancer cell plasticity (e.g. CD29, CD97, CD49c, ITGB5), published previously [30]. We also included markers that would allow for subtyping of stromal cells (e.g. CD90, Vimentin, and αSMA) and a spectrum of immune cell types (e.g. CD45, CD3, CD14, CD19). Additionally, to inspect the activation of tumor‐relevant signaling pathways at the level of protein, we included signaling hallmarks from TGF‐β/SMAD, NF‐κB, JAK–STAT, PI3K/AKT/mTOR, and Wnt signaling pathways. The activation of these signaling effectors is often involved in cancer cell plasticity and tumorigenesis (Fig. 1B).

This workflow resulted in 894 942 high‐quality single‐cell proteomic profiles from 26 samples. To allow for a comprehensive view of TNBC “cytome”, we generated two‐dimensional maps from our data using the automated dimensionality reduction algorithm t‐distributed stochastic neighbor embedding (tSNE). Most cells were of hematopoietic origin (CD45^+^), followed by stromal (CD90^+^) and cancer compartments (PanCK^+^; Fig. 1C). Information about other cell types or cell subsets is not available due to the limited number of markers that the current stage of technology allows. The presence of major cell types was additionally confirmed by manual gating (Fig. S1A).

### 3.2 Ki‐67‐positivity and lymph node involvement at the time of surgery as a proxy for patient outcome

We expected that the population of cancer cells in our TNBC cases will be globally heterogeneous, with some subpopulations more and some less abundant in aggressive tumors. To approach this question in an unbiased manner and to correlate cancer subpopulations with clinical parameters, we took advantage of a recently published automated and unsupervised machine learning algorithm RAPID [21]. This concept allows the identification of phenotypically distinct populations and determines whether they stratify patient survival. Due to the prospective design of this study that required freshly resected primary tumors, the long‐term clinical outcome data, including metastatic spread and survival, was not available at the time of analysis. We therefore tested and robustly validated ‘Ki‐67 positivity and lymph node ratio’ (LNR) at the time of the primary tumor surgery as a surrogate predictive and prognostic marker in TNBC, with minor modifications [31, 32, 33] (Fig. S1B). This approach enabled us to stratify patients solely according to their Ki‐67% and lymph node involvement, as both parameters are routinely collected during diagnosis and widely available. As a proof‐of‐concept study, we correlated the introduced Ki‐67 + LNR index with survival in two independent retrospective TNBC tissue microarray cohorts that we later used for histological validations (Fig. S1C,D). With good agreement between the predictive power of Ki‐67 + LNR index and survival in retrospective cohorts, we applied this index to our prospective single‐cell proteomic study.

### 3.3 Cancer subpopulations associated with clinical parameters

**Fig. 2:**
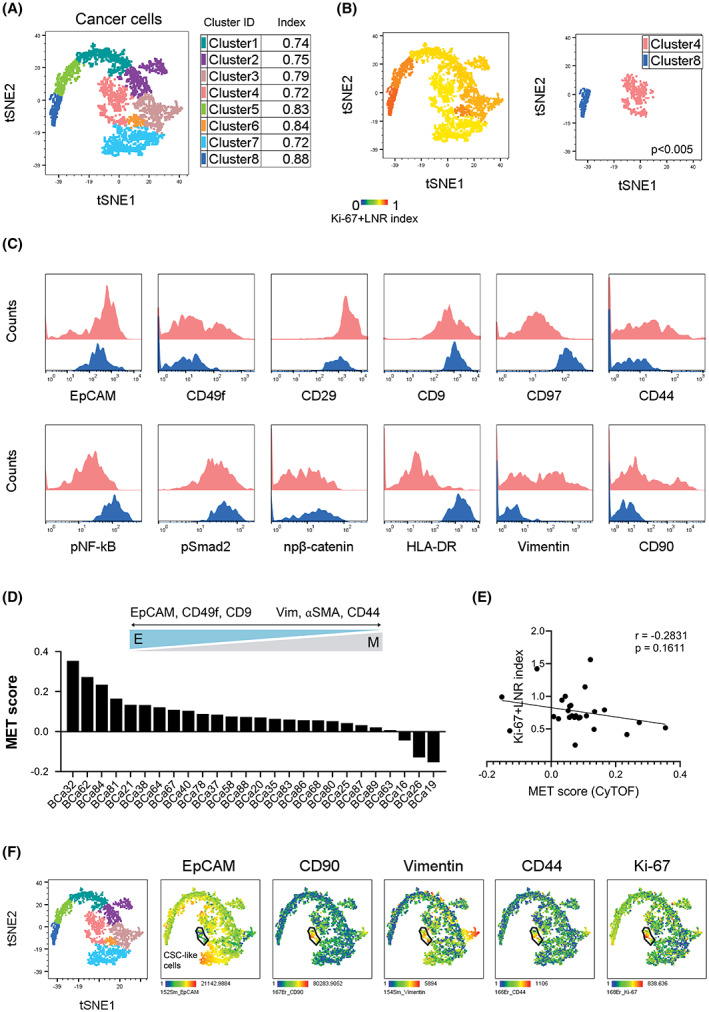
Complex analysis of cancer cells in TNBC tumors. (A) t‐SNE plot showing pooled cancer cells from 21 patients colored by FlowSOM clusters. (B) t‐SNE plot colored with Ki‐67 + LNR index (left), with depicted Ki‐67 + LNR‐high Cluster 8 (blue) and ‐low Cluster 4 (pink) populations in the right panel. (C) Histograms highlighting the difference in the expression of selected proteins in Ki‐67 + LNR‐high Cluster 8 (blue) and ‐low Cluster4 (pink). (D) Scheme depicting markers used for MET score computation (top) and plot showing calculated MET score for each patient (bottom). E, epithelial; M, mesenchymal. (E) Correlation of MET score with Ki‐67 + LNR index in the mass cytometry TNBC cohort (*r* = Spearman correlation coefficient). (F) t‐SNE plots highlighting identified CSC‐like population colored by the expression of selected antigens. CSC, cancer stem cells.

Simultaneous profiling of identically processed and preserved suspensions with mass cytometry eliminates the need for batch correction. We therefore applied modified algorithm employing FlowSOM clustering directly on cancer cells from our patients. FlowSOM provides an overview of how all markers are behaving on all cells, and detects subsets that might be otherwise missed. Using self‐organizing maps for data analysis, FlowSOM serves as both clustering and visualization tool [21, 24]. The identified FlowSOM clusters are then directly associated with Ki‐67 + LNR index. Using this data analysis approach, we observed eight phenotypically distinct populations of PanCK^+^ cells (Fig. 2A, Fig. S2A,B). Majority of analyzed tumors contained cells in all clusters (Fig. S2B, Table S2). These populations had distinct expression patterns of EMT/MET marker Vimentin, a number of cell adhesion molecules and integrins (CD29, CD49f, CD97, CD44, CD90), and signaling molecules (pNF‐κB and HLA‐DR; Fig. S2C,D). These TNBC subpopulations also differed in their Ki‐67 + LNR indices. While Cluster 8 had the highest calculated Ki‐67 + LNR index (blue; 0.88), Cluster 4 had the lowest (pink; 0.72) (Fig. 2B). Molecularly, cancer cells present in Cluster 8 displayed elevated expression of CD97, pNF‐κB, and HLA‐DR, and low CD90 and Vimentin levels (Fig. 2C). In contrast, Cluster 4 contained cells expressing low CD97, pNF‐κB and HLA‐DR levels, and high levels of CD49f, CD44, CD90, and Vimentin. The high expression of CD90 and Vimentin are frequently associated with epithelial‐mesenchymal transition (EMT). EMT and its reverse mesenchymal‐epithelial (MET) program are well‐known contributors to plasticity and heterogeneity of cancers, including TNBC. We therefore decided to predict the EMT‐MET phenotype of cancer cells in our patient cohort.

To assign cancer cells their EMT‐MET status in an unbiased manner, we calculated the mesenchymal‐to‐epithelial (MET) score for each cell and all patients based on selected epithelial and mesenchymal molecules. We selected Vimentin, αSMA, and CD44 as *bona fide* mesenchymal markers, and EpCAM, CD49f, CD9 as the representative of epithelial phenotype (Fig. 2D, [30]). The score ranged from −3 value representing fully mesenchymal phenotype, to +3 for fully epithelial phenotype, with hybrid phenotypes scoring around 0. We assessed that the cancer cells in 23 of 26 samples are highly EpCAM‐positive, with more than 50% cancer cells expressing this epithelial marker (average from all samples = 80.4%; 95% CI = 0.003) and exhibiting more epithelial phenotype based on their positive MET score. The MET score did not surpass 0.5 value, indicating that cells are, based on our classifier, in an intermediate or hybrid EMT/MET state, co‐expressing both epithelial and mesenchymal proteins (Fig. 2D). MET score negatively correlated with Ki‐67 + LNR index (Fig. 2E), suggesting that cancer cells favor hybrid EMT/MET phenotype endowing them with high plasticity and increased fitness, features that are required for the tumor development and progression.

Quite unexpectedly, we also identified Cluster 4 to be enriched for small population of both EpCAM^high^ and EpCAM^low^ cells with CD90/CD44/Vimentin/Ki‐67^high^ molecular profile, resembling proliferative cancer stem‐like (CSC) subpopulation (Fig. 2F). Such basal‐like CD44/CD90^+^ cells have been previously identified as tumorigenic and able to interact with monocytes and macrophages [34, 35].

### 3.4 Unprecedented heterogeneity of TNBC stromal compartment

**Fig. 3:**
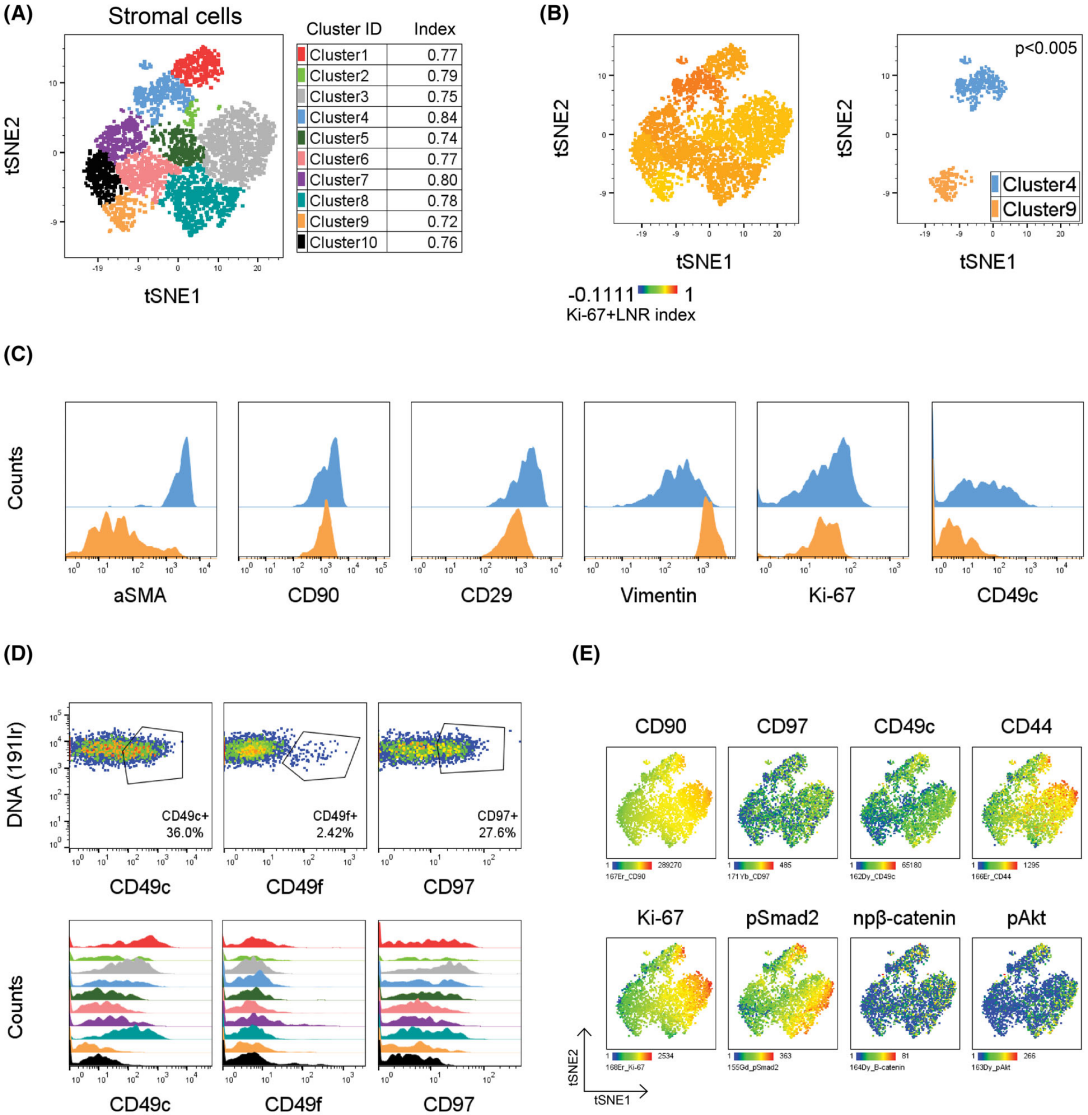
Identification of TNBC stromal compartment. (A) t‐SNE plot showing pooled stromal cells from 24 patients colored by FlowSOM clusters. (B) t‐SNE plot colored with Ki‐67 + LNR (left), with depicted Ki‐67 + LNR‐high Cluster 4 (blue) and ‐low Cluster 9 (orange) populations in right panel. (C) Histograms of selected stromal markers showing their expression in Cluster 4 (blue) and Cluster 9 (orange). (D) Subpopulations of CD49c‐, CD49f‐, and CD97‐positive stromal cells from 24 patients, visualized as dot plots (top panel) and as histograms across all 10 stromal clusters (bottom panel). (E) t‐SNE maps of stromal cells colored by expression profile of selected surface antigens and signaling molecules (npβ‐catenin – active, non‐phospho‐beta catenin).

In parallel, using similar unsupervised analysis in the CD90^+^/PanCK^−^/CD45^−^ stromal fraction, we molecularly dissected 10 stromal subsets of comparable abundance, present in 24 tumor specimens (Fig. S3A,B). Mirroring the situation in cancer compartment, these stromal subpopulations displayed diverse phenotypes with the most significant differences in the expression of CD90, αSMA, and CD29 – a set of well‐known cancer‐associated fibroblast (CAF) markers, surface integrin CD49c, adhesion G protein‐coupled receptor family member CD97, tetraspanin CD9, canonical TGF‐β signaling hallmark pSmad2, and proliferation surrogate Ki‐67 (Fig. S3C,D). Interestingly, these stromal clusters (Fig. 3A) showed similar Ki‐67 + LNR index with only minor difference in values, with the highest being Cluster 4 (blue, 0.84), and the lowest detected in Cluster 9 (orange, 0.72; Fig. 3B). This observation suggested that the identified stromal subsets do not stratify patients based on Ki‐67 + LNR index.

Compared to Cluster 9, cells in Cluster 4 (the highest Ki‐67 + LNR index) expressed high levels of αSMA, CD90, CD29, CD49c, and Ki‐67, also indicating an increased proliferative activity in this subpopulation (Fig. 3C). Because adhesion molecules and integrins, including CD49c, might play a functional role in tumor progression, we inspected whether these molecules are present also on stromal cells. We detected populations of CD49c‐, CD49f‐, and CD97‐positive cells present across clusters at different proportions (Fig. 3D). All three surface molecules were concomitantly co‐expressed, along with CD90 and CD44, with small “activated population” of cells expressing pSmad2, active b‐catenin, and phosphorylated Akt (Fig. 3E). This suggests that these stromal subsets co‐expressing signaling molecules might actively contribute to disease progression; however, precise functional experiments are needed. Although the stromal fraction did not differ in Ki‐67 + LNR index, we observed heterogeneity in stromal compartment and identified interesting subpopulations for further studies.

### 3.5 Validation of complex TNBC phenotypes with routine, low‐dimension immunohistochemistry

We next addressed the critical limitation of our study – the lack of long‐term clinical follow‐up in our freshly resected patient cohort – with an alternative strategy. We investigated the association between our mass cytometry protein hits with patient outcome in retrospective TNBC cohorts that included over 200 samples. We chose immunohistochemistry as a classical and routine procedure that does not require state‐of‐the‐art instrumentation and is widely used at clinical pathology departments for diagnostic purposes.

First, we in‐house assembled TNBC tissue microarray that included 108 primary tumors and if available, also matched lymph node metastases. For cross‐validation purposes, this cohort included archival tissue from 24 patients analyzed with mass cytometry. This sample set was then clinically annotated for lymph node involvement and, if available, also for survival.

**Fig. 4:**
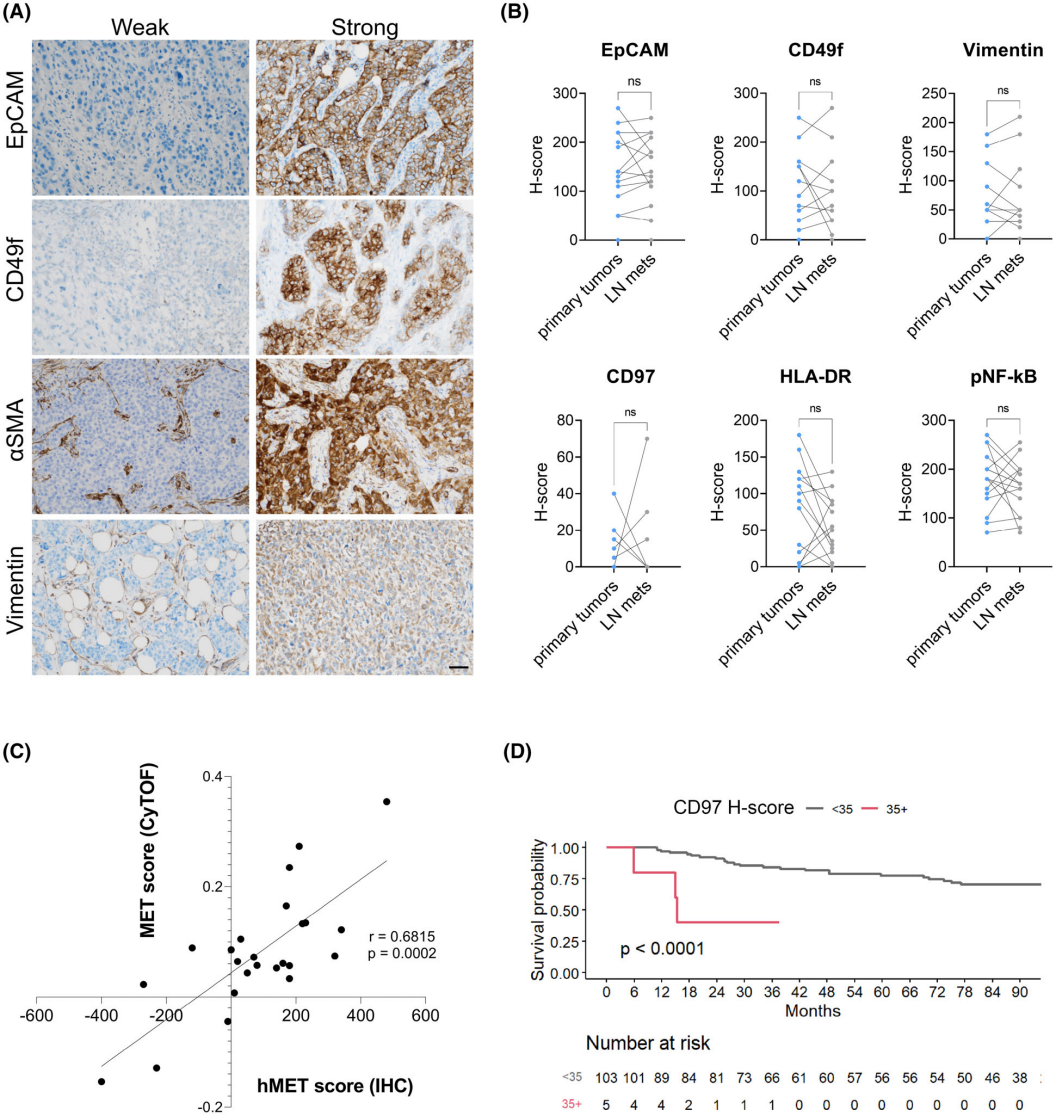
Immunohistochemistry staining of molecules identified by mass cytometry in TNBC cohort. (A) Representative immunostaining showing weak and strong EpCAM, CD49f, αSMA, and Vimentin stainings in TNBC specimens, ranked based on H‐score (paired t‐test). Sample is representative from the cohort of 108 primary tumor patients. Original magnification 200×, scale bar – 100 μm. (B) Paired analysis of EpCAM, CD49f, Vimentin, CD97, HLA‐DR, and pNF‐κB staining in cancer cells in primary TNBC tumors and matched lymph node metastases (*n* = 15) evaluated by paired *t*‐test. ns, not significant. (C) Concordance between mass cytometry MET score (CyTOF, *y*‐axis) and histology MET score (IHC, *x*‐axis) from the same cohort of 24 TNBC cases. *r* = Spearman correlation coefficient. (D) Kaplan–Meier curve showing overall survival of TNBC patients based on cancer cell CD97 expression. *n* = 108, logrank *P*.

Based on the unbiased outcomes from mass cytometry analysis, we selected proteins which expression significantly separated the Ki‐67 + LNR‐high and ‐low clusters, in both cancer and stromal compartments. These included CD97, HLA‐DR, pNF‐κB, and αSMA. Additionally, we included known markers of EMT/MET that were included in the cytometric panel, specifically EpCAM, CD49f, and Vimentin (Fig. 4A).

Percentage of positivity and staining intensity of selected markers in stromal and tumor cells for individual patients was determined using the semiquantitative H‐score method by a certified breast cancer pathologist in a blinded fashion. Although we were not able to reach statistical significance, this histopathological analysis revealed different expression patterns of EpCAM, CD49f, Vimentin, CD97, HLA‐DR, and pNF‐κB in lymph node metastases compared to matched primary tumors (Fig. 4B). These expression patterns varied from patient to patient and showed both increasing and decreasing trends in metastases relative to primary tumors, corroborating profound heterogeneity among the individual patients.

To approach this assessment in a similar way as done for the mass cytometry data, we classified the EMT/MET status of this retrospective histology cohort using histology MET score (hMET score). The hMET score was calculated from H‐score (0–300) of epithelial markers EpCAM and CD49f, and mesenchymal markers Vimentin and αSMA. Such hMET score extracted specifically from tumors that were analyzed with mass cytometry was in an agreement with the actual mass cytometry MET score (Fig. 4C). hMET score could be then, in simplified practice, used as a surrogate for mass cytometry MET score.

Out of all analyzed antigens, only the expression of EpCAM was significantly increased in tumors that disseminated into lymph nodes; the levels of EpCAM separated patients that had lymph nodes involved at the time of primary tumor resection from those that had not lymph node metastases present (Fig. S4A). Levels of HLA‐DR and pNF‐κB in the tumor fraction did not stratify patient survival (Fig. S4B). However, increased cancer cell expression of CD97 was significantly associated with worse overall survival, in concordance with our mass cytometry data (Fig. 4D). Although the correlation of stromal Vimentin with survival did not show significance, we observed an interesting trend between elevated stromal αSMA expression and worse overall survival (Fig. S4C).

## 4 Discussion

The recent revolution in single‐cell and spatial omics technologies revealed an unexpected level of TNBC heterogeneity. Despite this, the contribution of different tumor or stromal cell subsets to disease progression and therapy resistance remains unclear.

To understand how selected subpopulations and cell states shape tumor biology and influence the clinical outcome of TNBC patients, we established a single‐cell proteomics pipeline, allowing complex analysis of TNBC heterogeneity with mass cytometry. This approach allowed us to characterize TNBC “cytome” and associated diverse cancer and microenvironmental phenotypes with clinical state of 26 patients at the time of tumor surgery. While most single‐cell‐based TNBC studies focus on immune cells [36] or solely rely on transcriptomic data [15, 17, 18, 37], we used proteins as the ultimate readout.

Due to the prospective nature of our study, clinical outcome related to distant metastasis or patient survival was not available at the time of analysis. We therefore modified an alternative approach and introduced the Ki‐67 + LNR index that reflects Ki‐67% positivity and lymph node involvement at the time of surgery. Both of these parameters are relevant, standalone predictive and prognostic factors in TNBC, and their higher levels are associated with worse prognosis and more aggressive clinical features [31, 32, 33]. Nonetheless, there are some concerns about the reproducibility of the Ki‐67 assessment and its limited clinical utility [38]. Recent Ki‐67 consensus meeting established that Ki‐67 IHC does have clinical validity for the determination of prognosis in patients with early‐stage breast cancer and proposed several recommendations that can lead to precise analytical validity of Ki‐67 IHC determination namely careful preanalytical handling and standardized visual scoring [39]. These methodical criteria were met in our study that involved early‐stage TNBC patients (see Section 2).

Data analysis uncovered eight clinically distinct subsets of cancer cells, each with a specific protein signature. The cluster that significantly separated cell populations with high and low Ki‐67 + LNR index was enriched in HLA‐DR, pNF‐κB, and CD97. CD97 also significantly stratified patients based on their overall survival in our histology studies. CD97 is a member of G‐protein‐coupled receptor family with involvement in adhesion, migration, and cancer progression [40, 41]. CD97‐positive cancer cells associated with higher Ki‐67 + LNR index in patients and concomitantly expressed HLA‐DR – an MHC class II molecule, normally expressed by the antigen‐presenting cells, but also frequently identified in TNBC samples [42, 43], and pNF‐κB, a transcription factor implicated in TNBC proliferation and invasiveness with drug targeting potential [44, 45]. Our findings suggest that the identified population highly expressing CD97/HLA‐DR/pNF‐κB proteins might be functionally involved in TNBC progression, and clinically targeted in the future.

In addition, we showed that the cancer cells in TNBC tumors reside in a spectrum of hybrid EMT/MET states based on the introduced MET score, consisting of key epithelial and mesenchymal markers. Such hybrid EMT/MET phenotype embodies a major survival advantage: it maintains a highly plastic and tumorigenic state that can be dynamically polarized toward EMT or MET, thereby promoting cancer cell spread or homing and metastatic outgrowth at distant sites [5, 8, 11, 46, 47]. In our study, the hybrid EMT/MET profile of cancer cells was further confirmed by an independent immunohistochemistry using the hMET score. This scoring in the matched samples was associated with high Ki‐67 + LNR index and suggested that the hybrid phenotype may indeed drive tumor cell proliferation and metastatic lymph node colonization.

The stromal compartment of TNBC displayed a profound heterogeneity in cell surface and intracellular marker expression. We identified subsets of stromal cells co‐expressing high levels of αSMA, CD90, and CD29 that associated with high Ki‐67 + LNR index. This subpopulation resembled the profile of previously described myofibroblast‐like (αSMA^high^CD90^high^ FAP^high^) subsets of cancer‐associated fibroblasts (myCAFs) [17, 37, 48]. These myCAFs are functionally capable to initiate EMT of cancer cells, hence support their spread to distant organs [49]. The TNBC stroma was also enriched for cell subsets positive for surface integrins CD49f and CD49c, and adhesion G‐protein‐coupled receptor member CD97. While the role of CAF‐expressed integrins in breast cancer tumors has been previously suggested [50], their functional implications in the TNBC context remain unclear.

Our study comes with several specific limitations that are commonly associated with techniques requiring tissue dissociation and more generally with mass cytometry‐based approach. Firstly, although extensively optimized, the tissue dissociation protocol used in our laboratory may lead to underrepresentation of some cell types, including epithelial cells that are relatively fragile and can be destroyed during the dissociation process [51]. Secondly, this dissociation protocol can potentially alter the cell surface composition. Thirdly, pre‐selected antibodies in this panel can bias the phenotyping attempts. For example, we considered the hereby identified stromal cells as cancer‐associated fibroblasts, but we cannot exclude the possibility that CD90^+^ fraction contained also mesenchymal stromal cells or perivascular‐like cells. The part of the tumor tissue that was provided to us by the pathologists may not fully represent the composition of the entire tumor. Additionally, because of the prospective nature of our study, we lack information about clinical outcome (e.g. distant metastasis, overall survival) due to the short follow‐up period.

## 5 Conclusions

In this study, we established a workflow for complex investigation of TNBC at a single‐cell resolution and described phenotypically distinct cell subsets of tumor and stromal cells.

We introduced a novel clinically relevant Ki‐67 + LNR index that can be associated with described heterogeneous cell subpopulations, thus identify cells that might contribute to disease development. Moreover, we identified cancer cell CD97 level as a predictor of worse clinical outcome. Taken together, our findings shed a new light on the heterogeneity of treatment‐naïve TNBC, providing valuable resource for future research in both basic and translational settings.

## Conflict of interest

The authors declare no conflict of interest.

## Author contributions

BK collected and processed patient samples, stained samples, analyzed and interpreted the data, and wrote and revised the manuscript. RF analyzed and interpreted the data. DK, AV, and TK performed mass cytometry experiments and helped with data analysis. JS helped with data analysis. PO performed statistical analyses in r software. JN managed clinical samples. PF, JB, and RO performed IHC and scored IHC TMA. JR interpreted the data and revised the manuscript. KS conceptualized and designed the study, interpreted the data, revised the manuscript, and supervised the study. All authors read and approved the final version of this manuscript.

## Supporting information


**Fig. S1.** Gating strategy of mass cytometry data and Ki‐67+LNR index introduction. (A) Example of gating strategy showing the identification of live cells (CisPt‐) and three populations of interest in a representative sample (BCa83): PanCK+ epithelial cells, CD45+ immune cells and CD90+ stromal cells. (B) Plot showing the calculated Ki‐67+LNR index for each patient in TNBC cohort. (C) Kaplan‐Meier plot showing the relationship between survival probability and high/low Ki‐67+LNR index in a discovery cohort of archived TNBC patients that included samples used for mass cytometry measurement (n = 108). (D) Kaplan‐Meier plot showing the relationship between survival probability and high/low Ki‐67+LNR index in an independent, validation cohort of archived TNBC patients (n = 123).
**Fig. S2.** Unsupervised analysis of cancer cells in TNBC tumors. (A) t‐SNE map of cancer cells illustrating identified 8 clusters associated with Ki‐67+LNR index colored by FlowSOM clustering. (B) tSNE map colored by Ki‐67+LNR index ‐ left, Ki‐67+LNR index values in all clusters on histogram ‐ middle, contribution of cancer cells from patients (Sample ID) to identified clusters – right. (C) Histograms depicting expression of selected proteins in all cancer clusters. (D) Heatmap of normalized marker expression for different proteins in 8 clusters.
**Fig. S3.** Unsupervised analysis of TNBC stromal compartment. (A) t‐SNE analysis of stromal cells illustrating identified 10 clusters associated with Ki‐67+LNR index colored by FlowSOM clustering. (B) tSNE map colored by Ki‐67+LN index ‐ left, Ki‐67+LNR index values in all clusters on histograms ‐ middle, contribution of stromal cells from patients (Sample ID) to identified clusters ‐ right. (C) Histograms depicting expression of selected proteins in all stromal clusters. (D) Heatmap of normalized marker expression of different surface and intracellular proteins in 10 clusters.
**Fig. S4.** Immunohistochemistry staining in TNBC TMA. (A) Expression of selected proteins in primary tumors with detected lymph node metastasis (LN met positive) versus primary tumors without lymph node metastasis (LN met negative; total n = 107). (B) Overall survival of TNBC patients from TMA cohort stratified based on NF‐kB p65 and HLA‐DR staining (H‐score) in cancer cells. (C) Overall survival of TNBC patients from TMA cohort stratified based on αSMA and Vimentin expression (H‐score) in stromal cells.

**Table S1.** Clinical and histopathological parameters of TNBC patients.
**Table S2.** Percentage of cells present in cancer clusters across samples.

## Data Availability

The data that support the findings of this study are available from the corresponding author [ksoucek@ibp.cz] upon reasonable request.
