# Supplementary material for: Single‐cell protein profiling defines cell populations associated with triple‐negative breast cancer aggressiveness
Source: Mol Oncol. 2023 Jan 25;17(6):1024–40. doi: 10.1002/1878-0261.13365 (PMC10257414; doi:10.1002/1878-0261.13365)
Supplement: Supplementary file 1 — Fig. S1. Gating strategy of mass cytometry data and Ki‐67+LNR index introduction. (A) Example of gating strategy showing the identification of live cells (CisPt‐) and three populations of interest in a representative sample (BCa83): PanCK+ epithelial cells, CD45+ immune cells and CD90+ stromal cells. (B) Plot showing the calculated Ki‐67+LNR index for each patient in TNBC cohort. (C) Kaplan‐Meier plot showing the relationship between survival probability and high/low Ki‐67+LNR index in a discovery cohort of archived TNBC patients that included samples used for mass cytometry measurement (n = 108). (D) Kaplan‐Meier plot showing the relationship between survival probability and high/low Ki‐67+LNR index in an independent, validation cohort of archived TNBC patients (n = 123). Fig. S2. Unsupervised analysis of cancer cells in TNBC tumors. (A) t‐SNE map of cancer cells illustrating identified 8 clusters associated with Ki‐67+LNR index colored by FlowSOM clustering. (B) tSNE map colored by Ki‐67+LNR index ‐ left, Ki‐67+LNR index values in all clusters on histogram ‐ middle, contribution of cancer cells from patients (Sample ID) to identified clusters – right. (C) Histograms depicting expression of selected proteins in all cancer clusters. (D) Heatmap of normalized marker expression for different proteins in 8 clusters. Fig. S3. Unsupervised analysis of TNBC stromal compartment. (A) t‐SNE analysis of stromal cells illustrating identified 10 clusters associated with Ki‐67+LNR index colored by FlowSOM clustering. (B) tSNE map colored by Ki‐67+LN index ‐ left, Ki‐67+LNR index values in all clusters on histograms ‐ middle, contribution of stromal cells from patients (Sample ID) to identified clusters ‐ right. (C) Histograms depicting expression of selected proteins in all stromal clusters. (D) Heatmap of normalized marker expression of different surface and intracellular proteins in 10 clusters. Fig. S4. Immunohistochemistry staining in TNBC TMA. (A) Expression of selected pro [file MOL2-17-1024-s002.pdf]

Figure S1

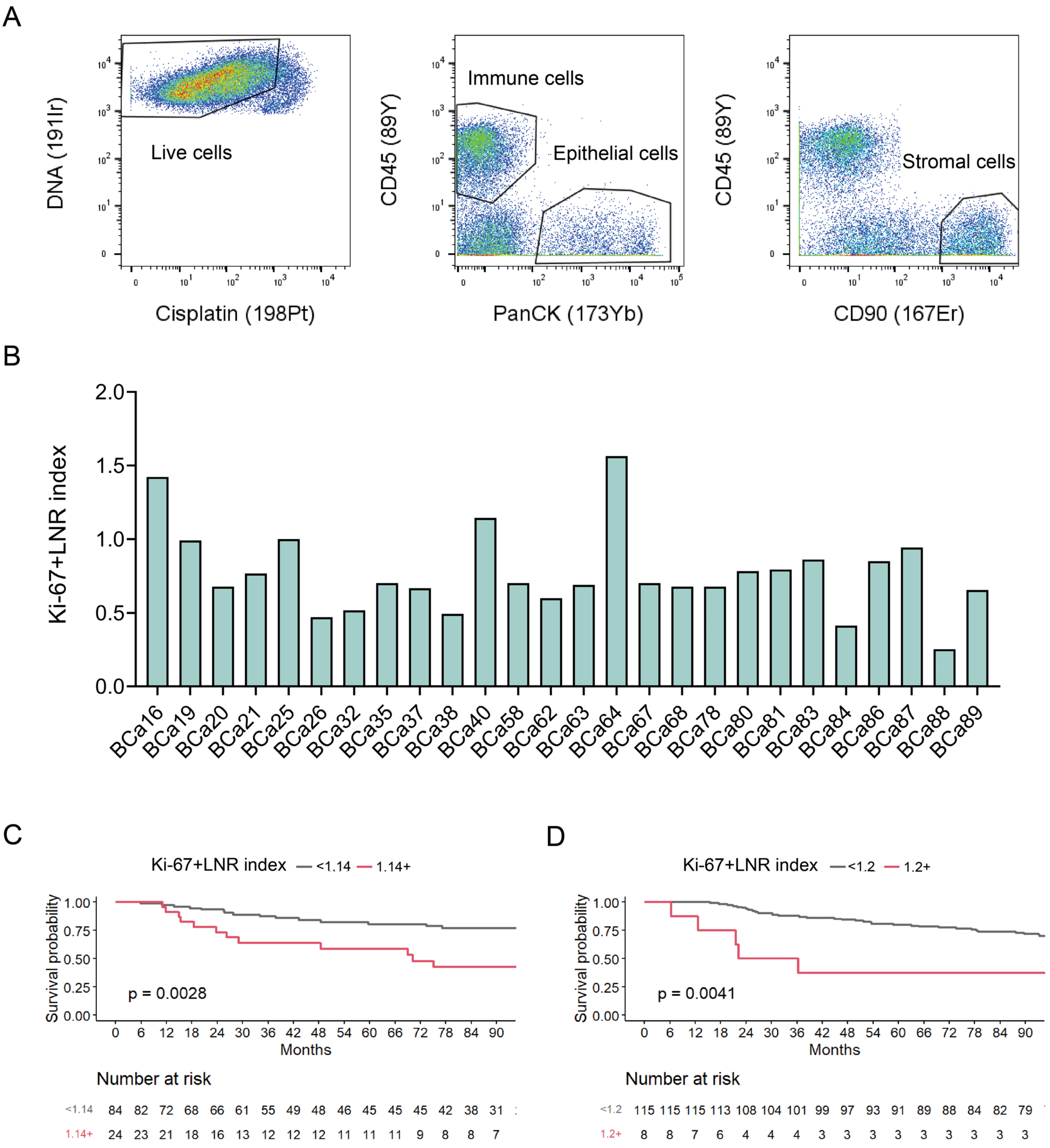

Figure S1: Gating strategy of mass cytometry data and Ki-67+LNR index introduction.
(A) Example of gating strategy showing the identification of live cells (CisPt-) and three populations of interest in a representative sample (BCa83): PanCK+ epithelial cells, CD45+ immune cells and CD90+ stromal cells. **(B)** Plot showing the calculated Ki-67+LNR index for each patient in TNBC cohort. **(C)** Kaplan-Meier plot showing the relationship between survival probability and high/low Ki-67+LNR index in a discovery cohort of archived TNBC patients that included samples used for mass cytometry measurement (n = 108). **(D)** Kaplan-Meier plot showing the relationship between survival probability and high/low Ki-67+LNR index in an independent, validation cohort of archived TNBC patients (n = 123).

Figure S2

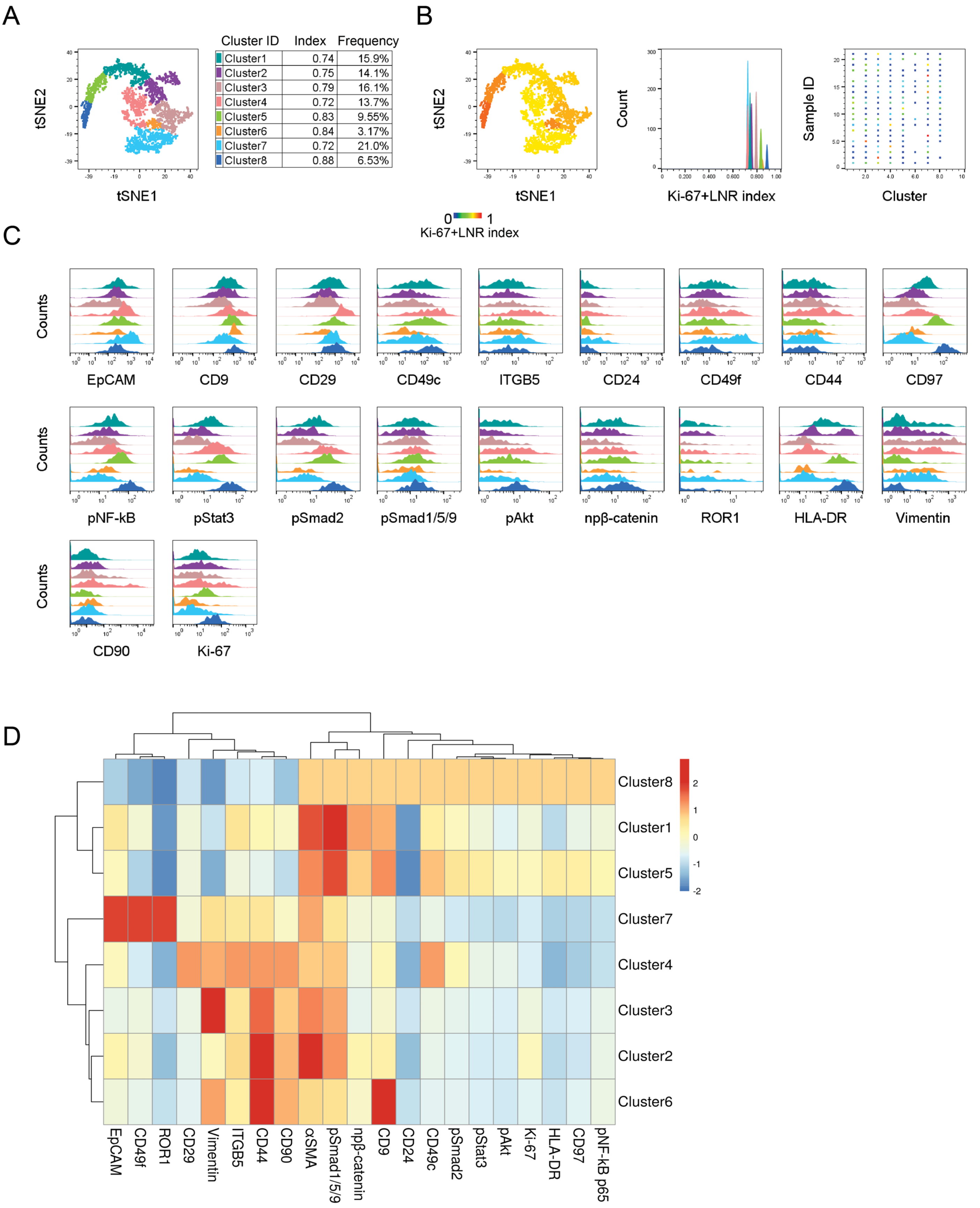

Figure S2: Unsupervised analysis of cancer cells in TNBC tumors.
(A) t-SNE map of cancer cells illustrating identified 8 clusters associated with Ki-67+LNR index colored by FlowSOM clustering. **(B)** tSNE map colored by Ki-67+LNR index - left, Ki-67+LNR index values in all clusters on histogram - middle, contribution of cancer cells from patients (Sample ID) to identified clusters – right. **(C)** Histograms depicting expression of selected proteins in all cancer clusters. **(D)** Heatmap of normalized marker expression for different proteins in 8 clusters.

Figure S3

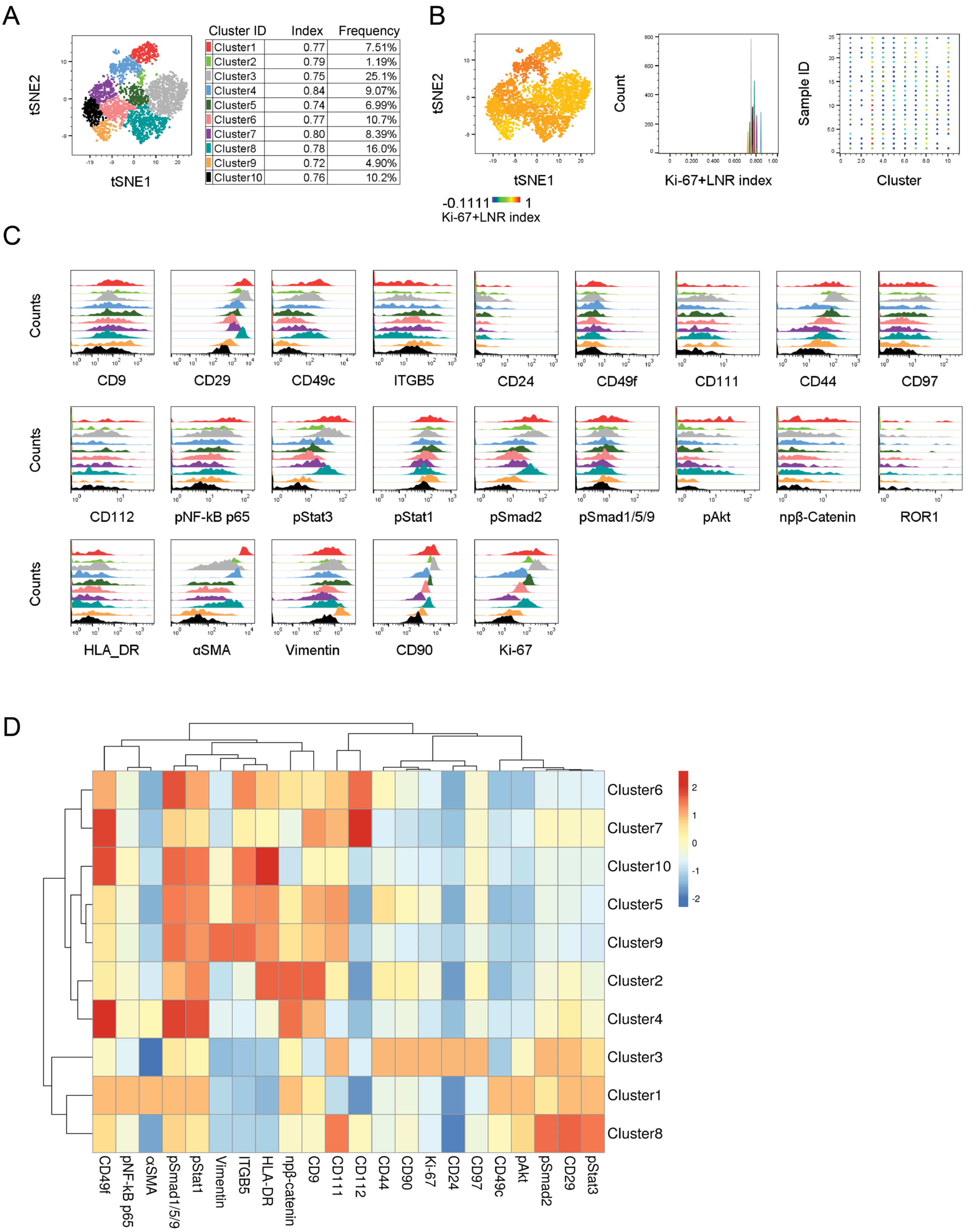

Figure S3: Unsupervised analysis of TNBC stromal compartment.
(A) t-SNE analysis of stromal cells illustrating identified 10 clusters associated with Ki-67+LNR index colored by FlowSOM clustering. **(B)** tSNE map colored by Ki-67+LN index - left, Ki-67+LNR index values in all clusters on histograms - middle, contribution of stromal cells from patients (Sample ID) to identified clusters - right. **(C)** Histograms depicting expression of selected proteins in all stromal clusters. **(D)** Heatmap of normalized marker expression of different surface and intracellular proteins in 10 clusters.

Figure S4

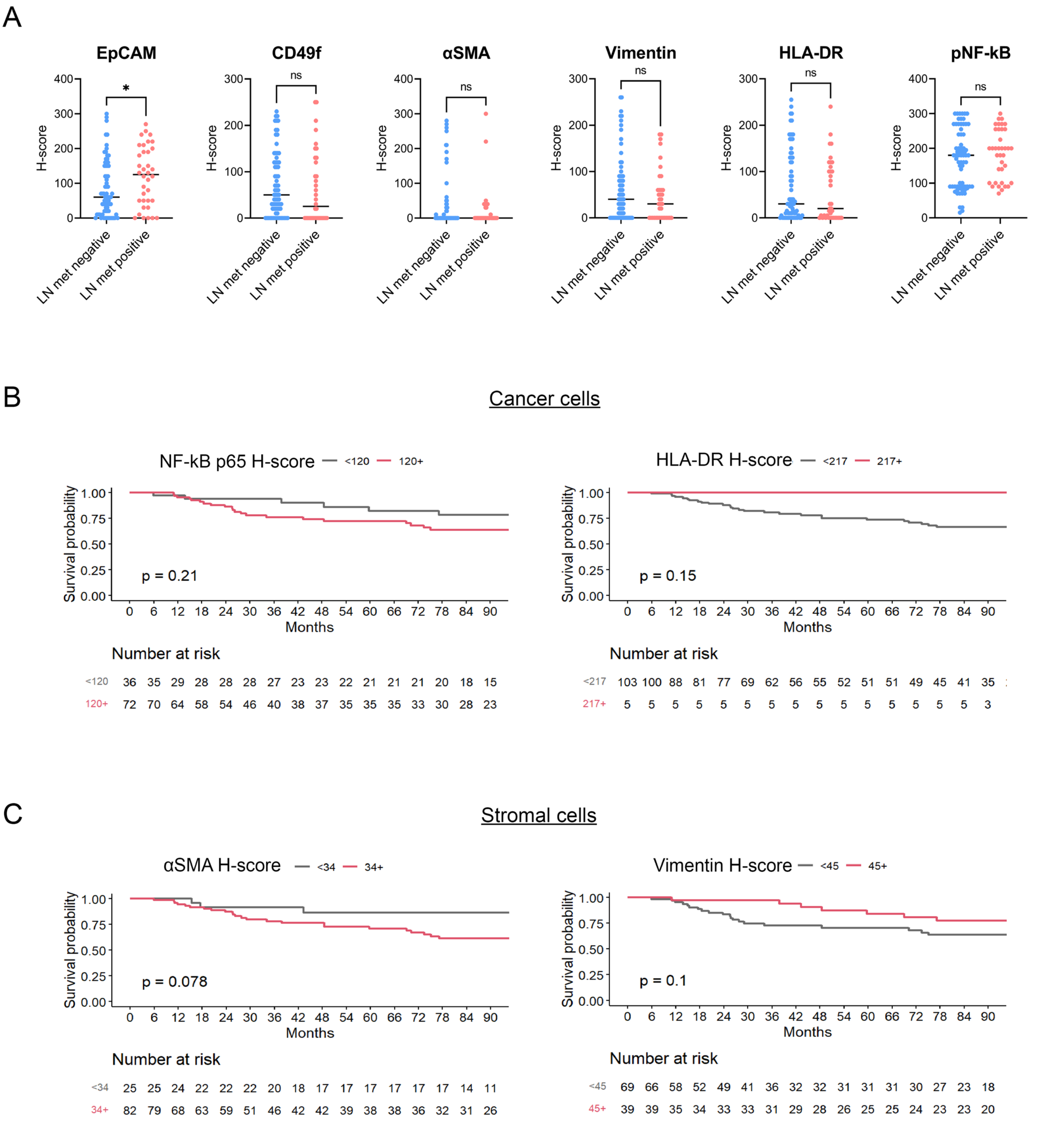

Figure S4: Immunohistochemistry staining in TNBC TMA.
(A) Expression of selected proteins in primary tumors with detected lymph node metastasis (LN met positive) versus primary tumors without lymph node metastasis (LN met negative; total n = 107). **(B)** Overall survival of TNBC patients from TMA cohort stratified based on NF-kB p65 and HLA-DR staining (H-score) in cancer cells. **(C)** Overall survival of TNBC patients from TMA cohort stratified based on αSMA and Vimentin expression (H-score) in stromal cells.
