# Supplementary material for: Single‐cell protein profiling defines cell populations associated with triple‐negative breast cancer aggressiveness
Source: Mol Oncol. 2023 Jan 25;17(6):1024–40. doi: 10.1002/1878-0261.13365 (PMC10257414; doi:10.1002/1878-0261.13365)
Supplement: Supplementary file 2 — Table S1. Clinical and histopathological parameters of TNBC patients. Table S2. Percentage of cells present in cancer clusters across samples. [file MOL2-17-1024-s001.docx]

**Supplementary table 1:** Clinical and histopathological parameters of TNBC patients

| Sample ID | age at diagnosis | histology | grade | pT | pN | positive lymph nodes | resected lymph nodes | M | Ki-67 (%) | Burstein subtype |
| --- | --- | --- | --- | --- | --- | --- | --- | --- | --- | --- |
| BCa16 | 68 | invasive carcinoma NST | 3 | 2 | 3a | 15 | 19 | 0 | 55 | BLIA |
| BCa19 | 73 | mixed metaplastic carcinoma | 3 | 3 | 1a | 1 | 18 | 0 | 81 | MES |
| BCa20 | 84 | invasive carcinoma NST | 3 | 2 | 0 |  |  | 0 | 59 | BLIS |
| BCa21 | 82 | invasive carcinoma NST | 3 | 2 | 1a | 3 | 17 | 0 | 51 | unspec. |
| BCa25 | 33 | invasive carcinoma NST | 3 | 1c | 0 |  |  | 0 | 87 | BLIA |
| BCa26 | 83 | metaplastic carcinoma | 3 | 3 | 0 |  |  | 0 | 41 | MES |
| BCa32 | 69 | invasive carcinoma NST | 3 | 1c | 0 |  |  | 0 | 45 | unspec |
| BCa35 | 49 | invasive carcinoma NST | 3 | 1c | 0 |  |  | 0 | 61 | BLIA |
| BCa37 | 27 | metaplastic carcinoma | 3 | 3 | 0 |  |  | 0 | 58 | BLIS |
| BCa38 | 83 | invasive carcinoma NST | 3 | 3 | 0 |  |  | 0 | 43 | unspec |
| BCa40 | 57 | invasive carcinoma NST | 3 | 2 | 2a | 6 | 12 | 0 | 56 | BLIS |
| BCa58 | 68 | invasive metaplastic carcinoma | 3 | 1c | 0 |  |  | 0 | 61 | N/A |
| BCa62 | 83 | carcinoma with apocrine differentiation | 3 | 2 | 1a | 2 | 14 | 0 | 40 | unspec |
| BCa63 | 36 | invasive carcinoma NST | 3 | 1c | 0 |  |  | 0 | 60 | unspec |
| BCa64 | 75 | invasive carcinoma NST | 3 | 3 | 1 | 1 | 1 | 0 | 49 | unspec |
| BCa67 | 66 | invasive carcinoma NST | 2 | 2 | 0 |  |  | 0 | 61 | N/A |
| BCa68 | 72 | invasive carcinoma NST | 3 | 1c | 0 |  |  | 0 | 59 | BLIA |
| BCa78 | 50 | invasive carcinoma NST | 3 | 1c | 0 |  |  | 0 | 59 | BLIS |
| BCa80 | 42 | invasive carcinoma NST | 3 | 2 | 0 |  |  | 0 | 68 | mixed basal |
| BCa81 | 68 | invasive carcinoma NST | 3 | 1c | 0 |  |  | 0 | 69 | unspec |
| BCa83 | 66 | invasive carcinoma NST | 3 | 2 | 0 |  |  | 0 | 75 | unspec |
| BCa84 | 67 | invasive carcinoma NST | 3 | 1c | 0 |  |  | 0 | 36 | BLIS |
| BCa86 | 56 | invasive carcinoma NST | 3 | 2 | 0 |  |  | 0 | 74 | BLIS |
| BCa87 | 70 | invasive carcinoma NST | 3 | 2 | 0 |  |  | 0 | 82 | unspec |
| BCa88 | 77 | invasive apocrine adenocarcinoma | 2 | 1c | 0 |  |  | 0 | 22 | LAR |
| BCa89 | 44 | invasive carcinoma NST | 3 | 2 | 0 |  |  | 0 | 57 | MES |

BLIA – basal-like immunoactivated, BLIS – basal-like immunosuppressed, MES – mesenchymal, LAR – luminal-androgen receptor, unspec – unspecified, NST – invasive carcinoma of no specific type

**Supplementary table 2:** Percentage of cells present in cancer clusters across samples

| **Sample ID** | **Cluster1** | **Cluster2** | **Cluster3** | **Cluster4** | **Cluster5** | **Cluster6** | **Cluster7** | **Cluster8** |
| --- | --- | --- | --- | --- | --- | --- | --- | --- |
| BCa16 | 4.66% | 6.68% | 11.20% | 6.43% | 0.60% | 4.50% | 0.95% | 0% |
| BCa19 | 1.61% | 1.82% | 22.70% | 1.24% | 1.79% | 0% | 0.68% | 1.75% |
| BCa20 | 1.79% | 3.04% | 4.44% | 10.80% | 1.19% | 11.70% | 6.53% | 0% |
| BCa21 | 1.43% | 2.02% | 4.09% | 20.30% | 0.60% | 0% | 3.54% | 0% |
| BCa25 | 4.48% | 20.90% | 0.89% | 1.04% | 4.48% | 0% | 0.14% | 5.68% |
| BCa32 | 0% | 0.61% | 1.07% | 0.83% | 0.30% | 0% | 20.50% | 0.87% |
| BCa35 | 11.80% | 8.10% | 2.13% | 0.21% | 8.36% | 0% | 0% | 8.73% |
| BCa37 | 3.76% | 3.24% | 3.73% | 20.10% | 2.09% | 0.90% | 0.41% | 0.44% |
| BCa40 | 6.27% | 9.92% | 6.57% | 3.11% | 1.79% | 17.10% | 0.68% | 0.44% |
| BCa62 | 9.14% | 1.01% | 1.42% | 4.56% | 11.60% | 0% | 3.81% | 6.11% |
| BCa64 | 7.35% | 0.61% | 0.53% | 1.24% | 21.80% | 0% | 0.54% | 16.20% |
| BCa67 | 10.90% | 5.47% | 0.71% | 6.43% | 7.76% | 1.80% | 0.14% | 6.55% |
| BCa78 | 8.42% | 3.04% | 2.49% | 1.66% | 8.66% | 0% | 4.35% | 9.61% |
| BCa80 | 0.72% | 1.21% | 4.26% | 5.19% | 1.19% | 0.90% | 13.70% | 0.87% |
| BCa81 | 2.51% | 1.82% | 1.42% | 3.32% | 7.16% | 6.31% | 7.76% | 14.00% |
| BCa83 | 6.63% | 5.67% | 4.80% | 0.62% | 7.46% | 12.60% | 2.18% | 7.42% |
| BCa84 | 0% | 0.20% | 1.07% | 2.90% | 0.30% | 0% | 19.20% | 1.75% |
| BCa86 | 8.78% | 9.92% | 6.57% | 1.24% | 1.49% | 3.60% | 1.90% | 1.31% |
| BCa87 | 0.54% | 10.30% | 3.73% | 1.66% | 0% | 0.90% | 11.20% | 0.44% |
| BCa88 | 8.42% | 2.23% | 2.31% | 3.53% | 10.40% | 0% | 0.82% | 16.60% |
| BCa89 | 0.72% | 2.23% | 13.90% | 3.53% | 0.90% | 39.60% | 0.95% | 1.31% |
| samples with cells present | 19/21 | 21/21 | 21/21 | 21/21 | 20/21 | 11/21 | 20/21 | 18/21 |
| STDEVA | 0.0383 | 0.0494 | 0.0535 | 0.0572 | 0.0544 | 0.0938 | 0.0627 | 0.0550 |
| CI | 0.0005 | 0.0007 | 0.0007 | 0.0008 | 0.0007 | 0.0013 | 0.0009 | 0.0008 |
